# Effects of Post-Process on the Microstructure and Mechanical Performance of an LPBF-Fabricated Fe-Based Alloy

**DOI:** 10.3390/ma19112262

**Published:** 2026-05-27

**Authors:** Zhijie Wang, Jiarong Xiao, Peitao Chen, Muyi Kuang, Defan Wu, Guojie Liu, Liqiao Wang, Quanquan Han

**Affiliations:** State Key Laboratory of Advanced Equipment and Technology for Metal Forming, School of Mechanical Engineering, Shandong University, Jinan 250061, China

**Keywords:** laser powder bed fusion, Fe-based alloy, post-process cooling, microstructure evolution, mechanical properties

## Abstract

A novel Fe-based alloy, designated as AMSD, was designed using a machine-learning-assisted high-throughput strategy, and it was successfully fabricated by laser powder bed fusion (LPBF) additive manufacturing without crack formation. This work systematically investigated the effects of post-process cooling rates on the microstructure and mechanical performance of the LPBF-fabricated AMSD alloy. After solution treatment at 1200 °C for 2 h, two cooling conditions, namely air cooling (AC) and water quenching (WQ), were applied, followed by aging at 500 °C for 24 h. It was found that the as-built (AB) alloy exhibited a typical cellular structure, epitaxial columnar grains, and a continuous intercellular segregation network. Post-processing eliminated the segregation network and promoted a more homogeneous microstructure with multiscale precipitates. Compared with AC condition, WQ preserved a finer and denser population of grain-boundary borides and achieved a superior strength–ductility balance, with a UTS of 1072 ± 15 MPa and an elongation of 18.2 ± 0.3% achieved. In contrast, the AC sample exhibited a higher UTS of 1436 ± 45 MPa but lower ductility. These results demonstrate that post-process cooling rates play a key role in regulating precipitate evolution and mechanical performance in LPBF Fe-based alloys.

## 1. Introduction

Laser powder bed fusion (LPBF) has emerged as one of the most promising and widely utilized metallic additive manufacturing (AM) technologies. By employing a focused high-energy laser beam to selectively melt metal powders in a layer-by-layer manner based on digital three-dimensional (3D) models [1], LPBF enables the fabrication of near-net-shape components with exceptional geometric complexity and highly tailored internal architectures [2,3,4]. Owing to these unique capabilities, LPBF has attracted significant attention and has been increasingly applied in high-performance fields such as aerospace, automotive, and biomedical engineering [5].

However, the LPBF process is characterized by extremely non-equilibrium metallurgical conditions, including rapid localized melting and solidification, ultrahigh cooling rates, and complex cyclic thermal histories caused by repeated laser scanning [6,7,8]. These characteristics often generate steep temperature gradients and significant residual stresses within the fabricated components. Consequently, crack-related defects and microstructural heterogeneities are prone to occur during the fabrication process [9,10,11]. For conventional Fe-based alloys, such as GH2132, these issues are particularly pronounced when processed by LPBF, often resulting in the formation of solidification cracks and other structural defects. These defects not only deteriorate the microstructural stability but also significantly compromise the mechanical performance of the fabricated components [12,13]. Therefore, appropriate post-processing treatments are often required to modify the microstructure, relieve residual stress, and improve the overall mechanical properties of LPBF-fabricated alloys.

Various post-processing strategies have been developed to improve the microstructure and mechanical performance of LPBF-fabricated alloys. Among them, heat treatment, hot isostatic pressing (HIP), and surface modification techniques are the most adopted approaches. Heat treatment is widely used to relieve residual stress, regulate phase transformations, and tailor precipitation behavior [14,15]. Hot isostatic pressing, on the other hand, can effectively eliminate internal pores and reduce microcracks through the combined action of high temperature and high pressure, thereby improving the structural integrity of the fabricated components [16,17]. In addition, surface treatments such as shot peening, and laser remelting are often applied to enhance surface quality and mechanical performance [18,19]. These post-processing techniques play a crucial role in optimizing the microstructure and improving the service performance of LPBF-fabricated alloys.

Solution heat treatment is widely employed to dissolve undesirable phases, homogenize elemental distribution, and reduce microstructural segregation formed during the rapid solidification process of LPBF [20,21,22]. After solution treatment, the cooling rate plays a critical role in determining the subsequent microstructural evolution and phase stability. Different cooling conditions, such as air cooling and water quenching, can significantly influence precipitation behavior, grain structure, and residual stress distribution, thereby affecting the mechanical performance of the alloy [22,23,24,25]. Therefore, understanding the influence of different cooling paths after solution treatment on the microstructure and mechanical properties of LPBF-fabricated Fe-based alloys is of great importance. However, most previous studies have mainly focused on process optimization, crack suppression, or post-treatment of existing alloy systems, while the coupling between alloy design and cooling-path-dependent microstructural evolution remains insufficiently understood, especially for newly developed Fe-based LPBF alloys. In particular, it is still unclear how different post-solution cooling routes affect the microstructure and mechanical response of a crack-resistant Fe-based alloy specifically designed for LPBF.

In this work, a novel Fe-based alloy (nominally designated as AMSD for ease of reference) was designed through a machine-learning-assisted high-throughput alloy design strategy and successfully fabricated by LPBF without obvious solidification cracking. The effects of two post-process cooling routes, namely air cooling (AC) and water quenching (WQ), on the microstructural evolution, crystallographic characteristics, tensile properties, and fracture behavior of the LPBF-fabricated AMSD alloy were systematically investigated.

## 2. Materials and Methods

### 2.1. Experimental Materials and LPBF Process

**Figure 1:**
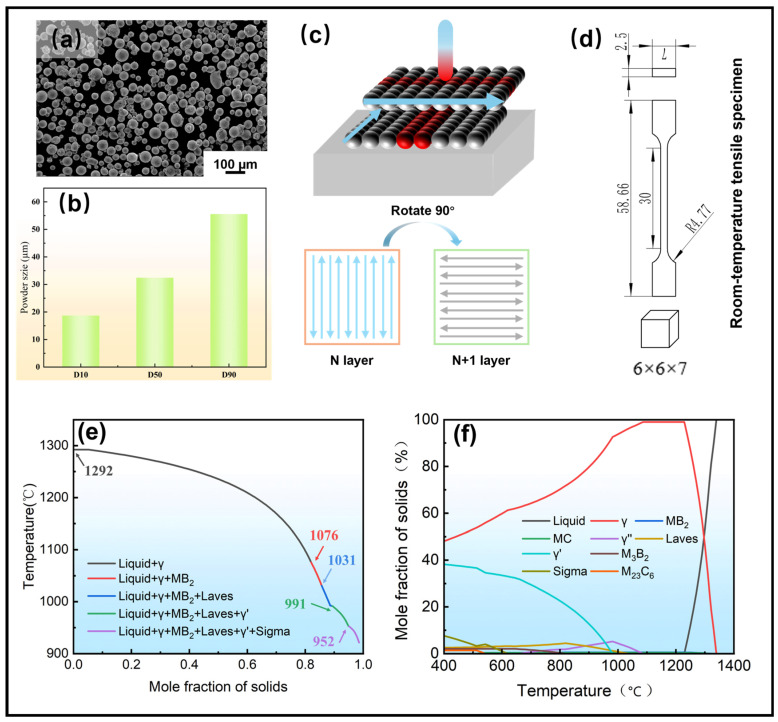
(**a**) The morphology of AMSD powder; (**b**) particle size distribution of AMSD powder; (**c**) scanning strategy; (**d**) dimensions of tensile specimen and cube specimen; (**e**) solidification curves and (**f**) simulation results of equilibrium solidification for AMSD alloy.

The AMSD metal powder used in this study was supplied by Avimetal Additive Manufacturing Technology (Beijing, China). Its chemical composition was determined by inductively coupled plasma optical emission spectrometry (ICP-OES) and infrared carbon–sulfur analysis, and the results are summarized in Table 1. The morphology of the AMSD powder is presented in Figure 1a, showing a high degree of sphericity with particle sizes ranging from 15 to 53 μm. The particle size distribution is shown in Figure 1b, where the characteristic diameters D10, D50, and D90 are 18.27, 32.47, and 55.57 μm, respectively.

All samples were fabricated using an M170H laser powder bed fusion (LPBF) system (Avimetal Additive Manufacturing Technology, Beijing, China) under a high-purity argon atmosphere with an oxygen content below 0.1%. The LPBF parameters were selected with reference to the work of Kuang et al. [12] on the conventional Fe-based superalloy GH2132. Accordingly, the laser power, hatch spacing, and layer thickness were kept constant at 190 W, 110 μm, and 40 μm, respectively, and no substrate preheating was applied. The laser scanning speed (LSS) was varied from 700 to 1500 mm/s to optimize the processing parameters. As illustrated in Figure 1c, a zigzag scanning strategy with a 90° rotation between successive layers was employed to reduce anisotropy. For parameter optimization and microstructural characterization, cubic specimens with dimensions of 6 mm × 6 mm × 7 mm were first fabricated at different scanning speeds. Subsequently, plate-shaped specimens measuring 60 mm × 8 mm × 2.5 mm were produced for tensile testing.

### 2.2. Thermodynamic Calculation

Thermodynamic calculations were performed using the Ni–Fe-based thermodynamic database implemented in JMatPro 7.0 software. The equilibrium phase diagram of the AMSD alloy was calculated based on its nominal chemical composition to predict the solidification behavior (Figure 1e) and phase evolution (Figure 1f), which guided the subsequent heat treatment design. The temperature range for the calculation was set from 400 °C to 1500 °C with a step size of 1 °C.

### 2.3. Heat Treatment

The heat treatment schedule was developed under the guidance of the equilibrium phase diagram (Figure 1f) [10]. Specifically, the LPBF-fabricated AMSD samples were first solution treated at 1200 °C for 2 h. After the solution treatment, the samples were cooled to room temperature using two different cooling methods, namely air cooling and water quenching, in order to investigate the influence of cooling rate on the microstructure and mechanical properties of the alloy. Subsequently, the samples were aged at 500 °C for 24 h. All heat treatment processes were conducted in a tubular furnace (NBD-T1700-80TIF, Henan NOBODY Material Technology Co., Ltd., Zhengzhou, China) under a high-purity argon atmosphere to minimize oxidation during the heat treatment.

### 2.4. Microstructure Characterization and Mechanical Properties Testing

To examine the defects and characterize the microstructure, cubic specimens were sectioned by wire cutting along the build direction. The cut surfaces were sequentially ground using SiC abrasive papers from 80# to 2500#, followed by mechanical polishing with 3 μm diamond suspension and 0.04 μm SiO_2_ colloidal suspension (Shanghai Yunbo Testing Technology Co., Ltd., Shanghai, China). The polished surfaces were first observed using an optical microscope (Beijing Yiguang Technology Co., Ltd., Beijing, China) to identify typical LPBF defects such as microcracks, lack of fusion (LOF), keyholes and pores, which were used to evaluate the processability of the alloy and determine the optimal processing parameters. Subsequently, the polished samples were chemically etched using a freshly prepared aqua regia solution (a volume ratio of HNO_3_:HCl = 1:3) for 8–15 s at room temperature to reveal the microstructure.

Microstructural characterization was performed using a field-emission scanning electron microscope (FE-SEM, JSM-7800F, JEOL, Akishima, Tokyo, Japan) equipped with electron backscatter diffraction (EBSD) and energy-dispersive X-ray spectroscopy (EDS). The EBSD measurements were conducted with an accelerating voltage of 15 kV and a beam current of 12 nA. The step size was set to 1 μm, and the scanning area was 800 × 800 μm^2^.

The geometry of the tensile specimen is shown in Figure 1d. Tensile tests were carried out using a Zwick-250 universal testing machine (ZwickRoell GmbH & Co. KG, Ulm, Germany) in accordance with the GB/T 228.1-2021. Metallic materials—Tensile testing—Part 1: Method of test at room temperature. Standards Press of China, Beijing, China, 2021. The strain was measured using an extensometer with a gauge length of 25 mm and the initial strain rate was set to 0.04 min^−1^, and the loading direction was perpendicular to the build direction. At least three specimens were tested for each condition, and the average values were reported as the ultimate tensile strength (UTS) and elongation (EL) of the AMSD alloy.

### 2.5. Alloy Design

**Figure 2:**
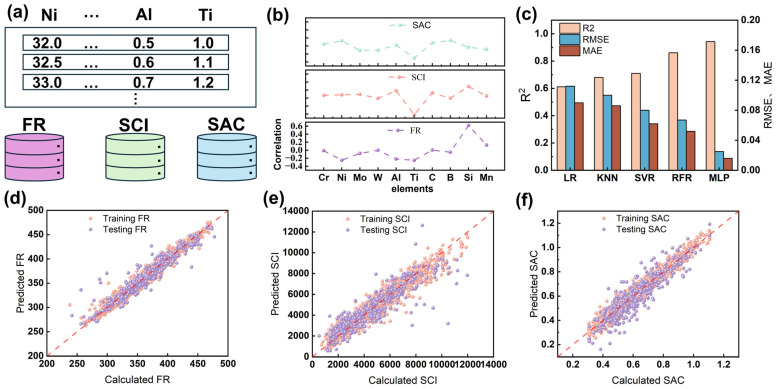
Machine-learning-assisted high-throughput design of the LPBF Fe-based alloy: (**a**) workflow of alloy design and dataset construction, the ellipsis indicates omitted intermediate data for schematic simplification; (**b**) Pearson correlation analysis of alloying elements with FR, SCI, and SAC; (**c**) performance comparison of different regression models; and predicted versus calculated results of the MLP model for (**d**) FR, (**e**) SCI, and (**f**) SAC, the red dashed line in the fitting scatter plot represents the y = x reference line.

Previous studies have demonstrated that machine-learning-assisted alloy design is a feasible and reliable approach for exploring complex composition–property relationships and accelerating alloy development [26,27,28,29]. On this basis, to develop a Fe-based alloy with low cracking susceptibility for LPBF, a machine-learning-assisted high-throughput alloy design strategy was established [30]. As schematically illustrated in Figure 2a, a large compositional dataset was generated by varying the principal alloying elements within a predefined design space. A composition–cracking-index dataset containing 3000 candidate alloy compositions was generated by high-throughput thermodynamic calculations within a predefined design space. Based on these thermodynamic results, three crack-related indicators, namely the freezing range (FR), solidification cracking index (SCI) [31], and strain age cracking index (SAC), were extracted to quantitatively evaluate the cracking susceptibility of the designed alloys [10]. These indicators were calculated according to Equations (1)–(3):

(1) $$FR=T_{l}-T_{S}$$

(2) $$SCI=\left|dT/d{(f}_{s}^{1/2})\right|$$

(3) $$SAC=dV_{f}^{\gamma }/dT,\;T\in (T_{\gamma }^{*},\;T_{s})$$

where $T_{l}$ and $T_{s}$ are the liquidus and solidus temperatures, respectively; T is the temperature; $f_{s}$ is the solid fraction; $V_{f}^{\gamma }$ is the volume fraction of the γ matrix; and $T_{\gamma }^{*}$ is the critical temperature at which the volume fraction of the γ phase reaches 0.7. Since FR, SCI, and SAC reflect different aspects of crack susceptibility during solidification and subsequent thermal evolution, simultaneous consideration of these three parameters enables a more comprehensive evaluation of alloy crack resistance than any single indicator alone. For each composition, FR, SCI, and SAC were calculated and used as the target outputs. Thus, alloy composition served as the input feature set, while FR, SCI, and SAC were used as the prediction targets. The dataset was divided into 80% training data and 20% testing data for model construction and evaluation.

To preliminarily reveal the compositional dependence of crack susceptibility, Pearson correlation analysis was conducted, and the corresponding correlation coefficients between alloying elements and the three crack-related indicators are summarized in Figure 2b. The Pearson correlation coefficient is defined as

(4) $$\begin{array}{cccc} & r_{xy}=\frac{\sum_{i=1}^{n}(x_{i}-\bar{x})(y_{i}-\bar{y})}{\sqrt{\sum_{i=1}^{n}(x_{i}-\bar{x}{)}^{2}}\sqrt{\sum_{i=1}^{n}(y_{i}-\bar{y}{)}^{2}}} & & \end{array}$$

where $r_{xy}$ denotes the correlation coefficient between variables x and y, $\bar{x}$ and $\bar{y}$ are their average values, and n is the number of samples. As shown in Figure 2b, the correlation coefficients vary markedly among different alloying elements and target indicators, indicating that FR, SCI, and SAC are governed by coupled compositional effects. Among all variables, Si exhibits the strongest positive correlation with FR, indicating that increasing Si tends to enlarge the freezing range. In contrast, Ti shows negative correlations with all three crack-related indicators, suggesting a generally beneficial role in suppressing crack susceptibility in the present alloy system. For SCI, no single alloying element exhibits an overwhelmingly dominant positive correlation; instead, SCI reflects the combined effects of multiple alloying elements, among which Ti shows the most pronounced negative correlation, while Si, Al, and C exhibit moderate positive correlations. Compared with FR and SCI, the correlation coefficients associated with SAC are generally weaker, indicating that SAC may be governed by more complex compositional interactions. Overall, these results suggest that optimization based on a single composition variable would be insufficient for rational alloy design.

To establish a surrogate model capable of capturing such nonlinear compositional effects, several representative regression algorithms, including linear regression (LR), K-nearest neighbors (KNN), support vector regression (SVR), random forest regression (RFR), and multilayer perceptron (MLP), were further evaluated. The predictive performance of these models was assessed using the coefficient of determination ($R^{2}$), root mean square error (RMSE), and mean absolute error (MAE) [32], and the corresponding comparison is shown in Figure 2c. It should be noted that Figure 2c presents the averaged testing performance over the three prediction targets, namely FR, SCI, and SAC, rather than the result for a single target. Among all evaluated models, MLP exhibited the best overall predictive performance, with the highest $R^{2}$ and the lowest prediction errors. For the machine-learning prediction, FR, SCI, and SAC were modeled using three independently trained multilayer perceptron (MLP) regressors rather than a single multi-output model. The hyperparameters of each MLP were optimized by grid search. The search space included hidden_layer_sizes = [(50,), (100,), (50, 50), (100, 50)], activation = {relu, tanh}, alpha = {0.0001, 0.001, 0.01}, and learning_rate_init = {0.001, 0.01}. The optimal models for FR and SCI both adopted a two-hidden-layer architecture of (50, 50) with tanh activation, while the SAC model used the same hidden-layer structure and activation function but a different optimal learning rate. The detailed prediction results of the MLP model for FR, SCI, and SAC are presented in Figure 2d–f, respectively. As shown in these parity plots, the predicted values for both the training and testing datasets are generally distributed close to the ideal y = x line, indicating good agreement between calculated and predicted results. The corresponding $R^{2}$ values for FR, SCI, and SAC reached 0.91, 0.79, and 0.75, respectively, indicating that the model provides the highest prediction accuracy for FR while maintaining satisfactory predictive capability for SCI and SAC. ese results demonstrate that the trained MLP model can effectively capture the nonlinear relationship between alloy composition and crack susceptibility, thereby making it a reliable surrogate model for subsequent inverse alloy design.

Based on the trained MLP model, multi-objective optimization was subsequently performed within the predefined compositional space to simultaneously minimize FR, SCI, and SAC. Herein, NSGA-III (nondominated sorting genetic algorithm III) was employed because it is well suited for multi-objective optimization with competing objectives and can effectively maintain diversity among Pareto-optimal solutions [32,33]. Rather than identifying a mathematically unique optimum, the optimization aimed to determine a candidate compositional region with low predicted cracking susceptibility by comprehensively balancing the three crack-related indicators. Since no explicit uncertainty propagation was incorporated in the present workflow, the optimization results were interpreted as a screening and prioritization tool rather than as an uncertainty-free exact solution. In this way, the design strategy avoids excessive bias toward a single cracking criterion and improves the robustness of the final alloy composition against different crack formation mechanisms. As a result, the optimized composition was finally determined and designated as AMSD, and its nominal composition is listed in Table 1.

This alloy was then selected for LPBF fabrication and subsequent post-process investigation. As demonstrated in Section 3, AMSD can be fabricated without obvious solidification cracking during LPBF, which confirms the effectiveness of the present alloy design strategy and provides a reliable basis for the subsequent post-process study.

## 3. Results and Discussion

### 3.1. Processability Analysis

**Figure 3:**
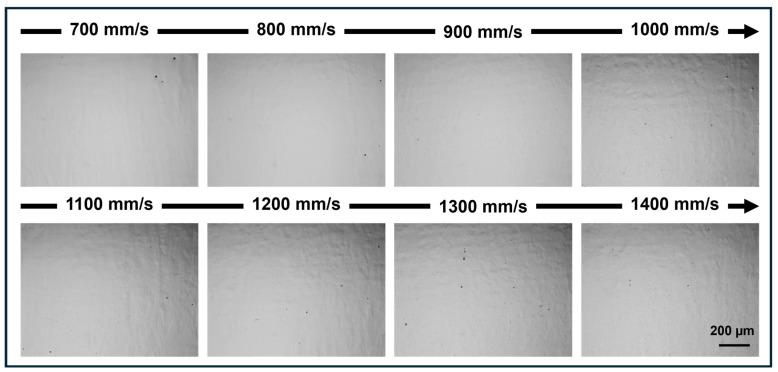
Optical microscopy (OM) images of laser powder bed fusion (LPBF)-fabricated AMSD alloy.

Figure 3 illustrates the optical microscopy (OM) morphologies of the as-built AMSD alloy prepared by LPBF at various laser scanning speeds. Notably, all samples are completely free of microcracks, explicitly demonstrating the outstanding structural integrity and processability of the investigated AMSD alloy. The defect formation is evidently dependent on the scanning speed: a limited number of pores are observed at a low speed of 700 mm/s, while minor lack of fusion (LOF) defects emerge when the speed reaches 1300 mm/s. Nevertheless, within the broad intermediate speed range, no obvious pores or defects are observed under OM, presenting a uniform and continuous surface morphology. This visual evidence intrinsically validates the excellent processability and wide processing window of the AMSD alloy. Based on the processability evaluation, the specimens used for the subsequent microstructural characterization and post-process study were fabricated at a laser scanning speed of 900 mm/s, which was selected by considering both the defect condition and the forming efficiency.

### 3.2. Microstructure Analysis

**Figure 4:**
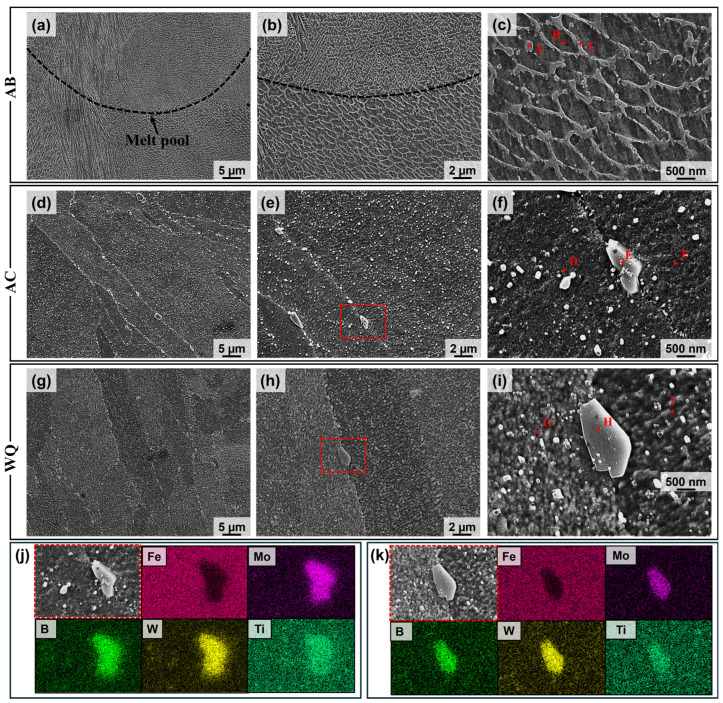
SEM micrographs showing the microstructural evolution of the (**a**–**c**) AB, (**d**–**f**) AC, and (**g**–**i**) WQ samples, along with the corresponding EDS elemental mapping (**j**,**k**) of the blocky precipitates. The dashed line indicates the melt-pool boundary, the dashed box marks the area enlarged in the corresponding high-magnification image, and A–I denote the representative marked features/locations.

Figure 4 shows the microstructural characteristics of the deposited alloy in the AB, AC, and WQ states. In the AB condition (Figure 4a–c), distinct arc-shaped melt-pool boundaries can be clearly observed, which are typical of layer-by-layer deposition and reflect the cyclic thermal history during solidification. At higher magnification, the matrix exhibits a very fine cellular substructure, indicating the rapid solidification nature of the deposition process. Meanwhile, a continuous network-like phase is observed along the intercellular regions or sub-grain boundaries. This feature suggests pronounced nonequilibrium solute redistribution during the terminal stage of solidification.

To further assess the micro-segregation behavior, EDS point analysis was performed on the representative locations marked in Figure 4c. As summarized in Table 2, Point B, located in the cellular interior, mainly represents the matrix and is enriched in Ni (38.35 wt.%) and Fe (34.70 wt.%). In contrast, Points A and C, located in the intercellular network region, show clear enrichment of segregating solute elements. In particular, Point C contains elevated levels of Cr (10.96 wt.%), Mo (4.03 wt.%), W (3.86 wt.%), Ti (5.68 wt.%), and C (9.94 wt.%), together with a reduced Ni content (28.76 wt.%). Such a compositional partitioning indicates that these alloying elements were rejected into the residual liquid during rapid solidification and subsequently concentrated in the intercellular regions. Considering the enrichment of C and refractory carbide/boride-forming elements (Mo, W, and Ti), this continuous network is more likely associated with complex non-equilibrium segregation products rather than discrete borides. No obvious blocky boride particles are observed in the as-built state, which may be attributed to the extremely high cooling rates during LPBF that suppress the formation of discrete boride phases. After post-deposition heat treatment, this segregation network is eliminated and discrete Mo-W-Ti-rich borides become clearly observable.

After post-deposition heat treatment, the microstructure changes significantly in both the AC and WQ conditions (Figure 4d–i). One notable feature is that the melt-pool boundaries become obscure and are no longer clearly distinguishable, suggesting that the heat treatment promotes elemental diffusion and reduces the chemical and structural heterogeneity inherited from the deposition process. More importantly, the continuous intercellular segregation network present in the AB state disappears after heat treatment. Instead, numerous fine precipitates are distributed more uniformly throughout the matrix and boundary regions. This transformation indicates that the non-equilibrium segregated network formed during deposition undergoes partial dissolution and redistribution during subsequent thermal exposure.

This microstructural evolution is important because continuous intercellular networks are generally unfavorable for mechanical integrity, especially when they are enriched in hard and brittle constituents. These networks can serve as preferential sites for strain localization and crack initiation. In contrast, the breakdown of the continuous network and the formation of fine dispersed precipitates are expected to reduce the continuity of weak interfaces and alleviate local stress concentration. Therefore, the post-deposition heat treatment is considered effective in modifying the as-deposited nonequilibrium microstructure into a more homogenized and potentially more damage-tolerant state.

In addition to the fine precipitates, coarse irregular blocky particles are also found in both AC and WQ samples, as highlighted in Figure 4e,h. The enlarged images in Figure 4f,i show that, although both AC and WQ samples contain blocky borides with similar phase characteristics, their local microstructures are different. The AC sample exhibits relatively coarser and less dense surrounding precipitates, whereas the WQ sample retains a finer and denser particle distribution, indicating that rapid quenching suppresses further coarsening, implying that the cooling medium has a limited effect on their overall morphology and stability. To clarify their chemical nature, EDS elemental mapping was performed on the selected coarse particles. As shown in the bottom panels of Figure 4j,k, these particles are strongly enriched in Mo, W, Ti, and B, while Fe is relatively depleted. This compositional signature strongly suggests that the coarse blocky precipitates are refractory-element-rich borides, which is consistent with previous reports that borides in Ni-based superalloys are commonly enriched in Mo and W and can exist as micrometer-scale grain-boundary precipitates [34,35].

Notably, although AC and WQ represent different cooling routes, Mo-W-Ti-rich borides are observed in both conditions. This indicates that the presence of the boride phase is consistent with its thermodynamic stability under the present post-processing conditions. However, this thermodynamic phase stability should be distinguished from the subsequent morphological evolution of the borides, which is mainly governed by kinetic effects. Although the boride phase remains stable, its growth and coarsening are still controlled by diffusion during cooling. As discussed later in Section 3.5, the slower cooling rate in AC provides more time for diffusional coarsening, whereas rapid quenching in WQ restricts diffusion and therefore preserves a finer boride distribution.

### 3.3. Crystallographic Orientation and Grain Boundary Characteristics

**Figure 5:**
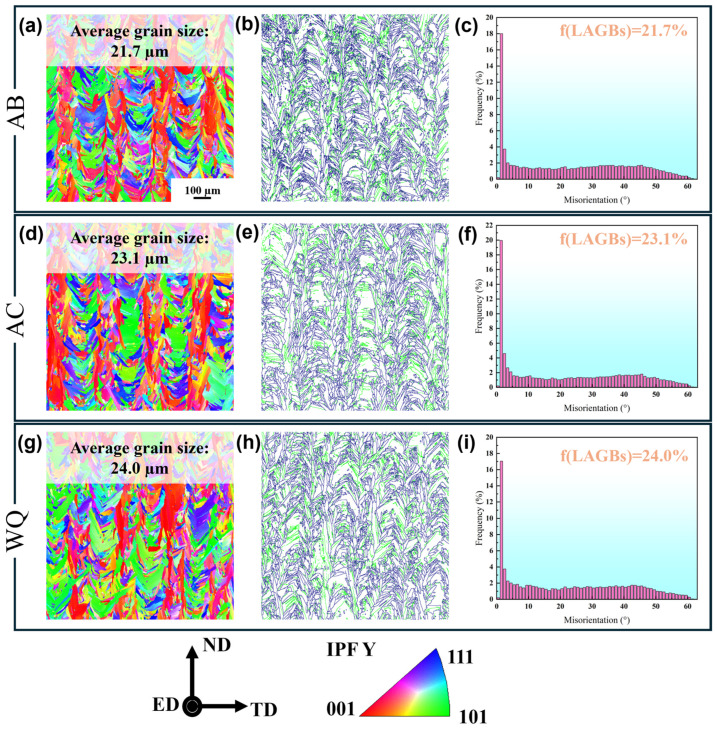
EBSD characterization of the AB, AC, and WQ samples: (**a**,**d**,**g**) IPF orientation maps; (**b**,**e**,**h**) grain-boundary maps, where the blue and green lines represent HAGBs and LAGBs, respectively; (**c**,**f**,**i**) misorientation angle distributions of AB, AC and WQ samples.

Figure 5 presents the EBSD inverse pole figure (IPF) maps, grain boundary character distributions, and misorientation histograms of the alloy in the AB (Figure 5a–c), AC (Figure 5d–f), and WQ (Figure 5g–i) states. In all three conditions, the microstructure is dominated by elongated columnar grains aligned approximately parallel to the building direction, indicating that the grain morphology established during deposition is largely retained after post-deposition heat treatment. Such columnar grains are characteristic of additively manufactured alloys and originate from competitive epitaxial growth under a steep thermal gradient along the build direction [21,36].

The average grain size exhibits only a slight increase after heat treatment, from 21.7 ± 0.32 μm in the AB sample to 23.1 ± 0.45 μm in the AC sample and 24.0 ± 0.24 μm in the WQ sample based on the analysis of approximately 3500 grains for each condition. This limited variation indicates that no pronounced grain coarsening occurs during either cooling route. The nearly unchanged grain size after post-deposition heat treatment can be understood from the limited grain-boundary mobility in the present alloy system. On the one hand, refractory substitutional solute elements such as Mo, W, and Ti are expected to exhibit relatively sluggish diffusion in the matrix and may interact with migrating grain boundaries, thereby exerting a solute-drag effect. On the other hand, dispersed secondary phases formed after heat treatment may also hinder grain-boundary migration during thermal exposure [37,38,39,40,41]. Therefore, although post-deposition heat treatment significantly modifies the local nonequilibrium segregated microstructure, its influence on the overall grain framework remains limited. As a result, although post-deposition heat treatment significantly modifies the local nonequilibrium segregated microstructure and eliminates the continuous segregation network inherited from the as-built state, it does not provide sufficient boundary mobility to induce pronounced grain coarsening. This interpretation is further supported by the retained high fraction of LAGBs and the persistence of columnar grains after both AC and WQ treatments, which together indicate that recovery may occur locally, whereas substantial recrystallization and grain growth remain limited.

The grain boundary character distribution further reveals a high fraction of low-angle grain boundaries (LAGBs, misorientation < 15°) in all samples. The measured LAGB fractions are 39.95% for AB, 43.27% for AC, and 41.24% for WQ. These values indicate that a substantial number of sub-grain boundaries and dislocation-derived internal interfaces are retained after heat treatment. Moreover, the LAGB fraction does not decrease after post-treatment; instead, it remains at a comparably high level, suggesting that the present heat-treatment conditions are insufficient to induce substantial recrystallization or to fully eliminate the as-deposited substructure [39,40,42,43]. The slightly higher LAGB fraction in the AC sample may reflect a certain degree of boundary rearrangement during thermal exposure, although the overall differences among the three conditions remain limited.

In addition, the boundary maps show that the internal boundary network is still mainly distributed within the columnar grains, rather than being replaced by newly formed equiaxed recrystallized grains surrounded by high-angle boundaries. This observation is consistent with the limited change in grain size and further confirms that the post-deposition treatments do not fundamentally reconstruct the grain morphology, since complete recrystallization is typically accompanied by the replacement of columnar subgrain structures by equiaxed grains and other clear recrystallization features such as cell disappearance and grain coarsening [39]. Instead, their major role appears to be the homogenization of local chemical segregation and the redistribution of secondary phases, as discussed in Figure 4.

**Figure 6:**
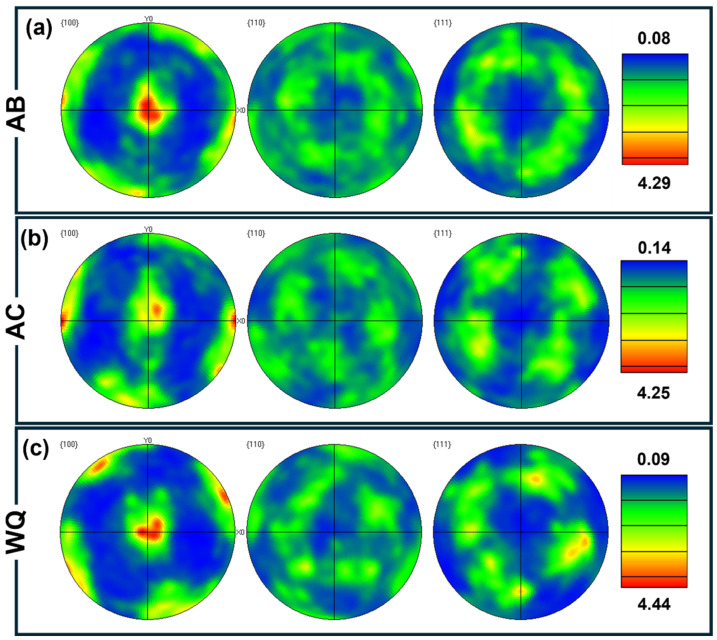
Crystallographic texture of the specimens under different heat-treatment conditions: (**a**) pole figures of the specimen AB; (**b**) pole figures of the specimen AC; (**c**) pole figures of the specimen WQ.

To further clarify the crystallographic orientation evolution, the {100}, {110}, and {111} pole figures are presented in Figure 6. The AB sample shows a pronounced preferential <100> fiber texture along the building direction, which is a typical feature of directional solidification in additive manufacturing. This texture arises from competitive epitaxial growth, where grains favorably oriented with respect to the maximum thermal gradient preferentially survive during layer-by-layer solidification.

After heat treatment, both AC and WQ samples still preserve similar overall pole figure characteristics, and no obvious texture randomization or recrystallization-induced orientation reconstruction is observed. This indicates that the dominant crystallographic framework established during deposition remains stable during subsequent heat treatment. Combined with the nearly unchanged grain size and the retained high fraction of LAGBs, the pole figure results further support the conclusion that both AC and WQ treatments lead to only limited recovery and recrystallization.

Overall, the EBSD results demonstrate that post-deposition heat treatment has little effect on the global grain morphology, grain size, and texture of the alloy, even though it significantly alters the local segregated microstructure. The retained columnar grains, high LAGB fractions, and preserved <100>-type texture collectively indicate that the primary role of the present heat treatments is the modification of local nonequilibrium microstructural features rather than reconstruction of the grain structure.

### 3.4. Mechanical Properties and Structure–Property Correlation

**Figure 7:**
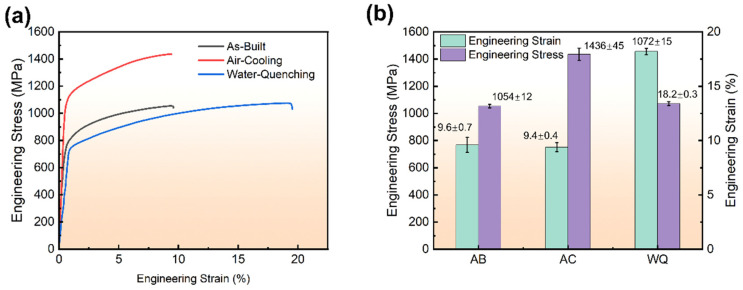
Tensile properties of the specimens under different heat-treatment conditions: (**a**) engineering stress–strain curves of the AB, AC and WQ specimens; (**b**) comparison of engineering stress and engineering strain for the AB, AC and WQ specimens.

Figure 7 shows the engineering stress–strain curves (Figure 7a) and the corresponding tensile properties (Figure 7b) of the AMSD alloy under the AB, AC, and WQ conditions. The AB sample exhibits an ultimate tensile strength (UTS) of 1054 ± 12 MPa and an elongation of 9.6 ± 0.7%. After air cooling, the tensile strength increases markedly to 1436 ± 45 MPa, while the elongation remains at 9.4 ± 0.4%, indicating a pronounced strengthening effect accompanied by a slight reduction in ductility. In contrast, the WQ sample exhibits a UTS of 1072 ± 15 MPa, which is close to that of the AB state, but its elongation increases substantially to 18.2 ± 0.3%. These results indicate that AC is more effective in maximizing strength, whereas WQ provides a much more favorable strength–ductility balance.

**Figure 8:**
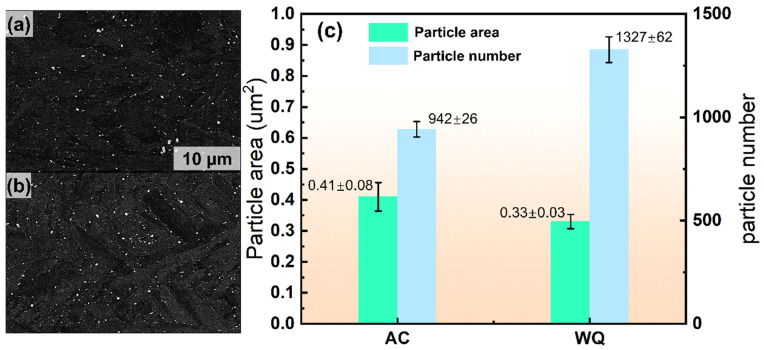
BSE images of AC (**a**) and WQ (**b**) samples; (**c**) the corresponding statistical analysis of particle area and number for the discrete grain boundary precipitates in the AC and WQ samples.

To better understand the origin of this mechanical divergence, quantitative analysis based on backscattered electron (BSE) images was performed on the discrete grain-boundary borides in the AC and WQ samples, as shown in Figure 8a,b. The statistical results (Figure 8c) reveal a clear difference in particle morphology and distribution between the two conditions. In the AC sample, the average particle area reaches 0.41 ± 0.08 μm^2^, with a particle number of 942 ± 26. By comparison, the WQ sample contains a significantly larger number of finer particles, with an average area of 0.33 ± 0.03 μm^2^ and a particle number of 1327 ± 62. This trend suggests that, although the boride phase remains stable in both cooling conditions, the slower cooling rate in the AC condition allows more extensive diffusional coarsening of the grain-boundary borides, whereas rapid quenching in the WQ condition suppresses further particle growth and preserves a finer distribution. Interfacial energy should also be considered in interpreting the coarsening of grain-boundary borides. In capillarity-driven ripening, the reduction in total interfacial area provides the thermodynamic driving force for particle growth [44,45]. Although the exact boride/matrix interfacial energy cannot be determined in the present work, recent studies on related metallic interfaces suggest that values on the order of $10^{-1}$–$10^{0}$ J/m^2^ are physically reasonable for less coherent complex interfaces [46]. Thus, interfacial energy provides the driving force for coarsening, while the cooling path controls its kinetic extent. In addition, the finer borides retained in the WQ condition may also contribute to grain-boundary stabilization. Based on recent analytical and AM-superalloy studies of particle pinning, the corresponding pinning pressure is estimated to be on the order of $10^{-2}$–$10^{-1}$ MPa [47,48], which is lower than the capillarity term but still non-negligible. Therefore, the capillarity term and the pinning effect should be regarded as competing contributions in the post-treatment microstructural evolution.

The fragmentation of the continuous brittle network observed in the AB state is an important microstructural change for both post-treated conditions. As discussed in Figure 4, post-deposition heat treatment eliminates the continuous intercellular segregation network and replaces it with discrete secondary phases, thereby reducing the continuity of brittle paths along internal boundaries. This microstructural modification is expected to alleviate premature crack initiation associated with the as-built non-equilibrium microstructure and thus provides a common basis for the improved mechanical stability of both AC and WQ samples relative to the AB condition. However, the distinct tensile responses of AC and WQ indicate that the morphology of grain-boundary borides alone cannot fully account for the observed difference in strength and ductility. In the AC sample, the much higher tensile strength suggests the presence of a stronger overall strengthening state, which may be associated not only with grain-boundary particle evolution but also with additional strengthening contributions from the matrix. By contrast, the WQ sample, which retains a finer and more numerous grain-boundary particle population, exhibits much greater elongation, implying a microstructure with a higher capacity for plastic strain accommodation. Therefore, the superior ductility of the WQ sample is mainly attributed to its finer and less-coarsened grain-boundary boride. Compared with the coarser borides in the AC sample, the finer and more uniformly distributed boundary precipitates in WQ are expected to reduce local stress concentration and delay crack initiation, thereby allowing greater plastic strain accommodation before failure; this results in the enhanced ductility of the WQ sample.

From a deformation perspective, coarser grain-boundary borides in the AC condition may contribute to local stress concentration more readily than the finer and more dispersed particles in the WQ condition, which is unfavorable for tensile ductility. This interpretation is consistent with previous studies showing that coarse grain-boundary borides or other brittle second-phase particles can intensify local stress concentration and facilitate microcrack initiation, thereby deteriorating tensile ductility [23,49]. Conversely, the finer grain-boundary particle distribution in the WQ sample is expected to weaken the severity of local boundary stress concentration and delay the formation of microcracks, thereby allowing greater uniform plastic deformation. At the same time, the lower strength of WQ relative to AC suggests that the matrix hardening state after quenching is less pronounced than that developed during air cooling.

Overall, the mechanical results demonstrate that post-deposition heat treatment effectively overcomes the detrimental effect of the continuous brittle segregation network inherited from the AB state, while the subsequent cooling path determines the balance between strength and ductility. Air cooling favors a higher strength level, whereas water quenching preserves a finer grain-boundary boride structure and achieves significantly improved tensile elongation. Thus, the mechanical response of the AMSD alloy is controlled by the combined effects of segregation elimination, grain-boundary particle evolution, and the overall strengthening state of the matrix.

### 3.5. Possible Strengthening Mechanisms

As shown in Figure 7a, the AC and WQ samples exhibit significantly different mechanical responses. The yield strength increases from 727 ± 25 MPa in the WQ state to 1098 ± 32 MPa in the AC state, corresponding to an increase of approximately 370 MPa. To analyze the possible strengthening mechanism, the yield strength ($\sigma_{ys}$) can be expressed as:

(5) $$\sigma_{ys}=\sigma_{0}+\sigma_{GB}+\sigma_{SS}+\sigma_{DIS}+\sigma_{PRE}$$

where $\sigma_{0}$, $\sigma_{GB}$, $\sigma_{SS}$, $\sigma_{DIS}$, and $\sigma_{PRE}$ denote the intrinsic strength, grain-boundary strengthening, solid-solution strengthening, dislocation strengthening, and precipitation strengthening, respectively.

Based on the microstructural characterization (Figure 5), the contributions from grain-boundary strengthening and dislocation strengthening are unlikely to differ substantially between the AC and WQ samples. Specifically, the two samples exhibit comparable average grain sizes and nearly identical fractions of low-angle grain boundaries (LAGBs), suggesting a limited difference in Hall–Petch strengthening and substructure-related strengthening. In addition, the average KAM values are 0.30° for AC and 0.35° for WQ, indicating only a slight difference in local lattice distortion and geometrically necessary dislocation (GND)-related strain storage. Since the WQ sample shows a slightly higher KAM value but a much lower yield strength, the pronounced strength increase in the AC sample is unlikely to originate mainly from dislocation strengthening. Consequently, the strengthening contribution arising from $\sigma_{GB}$ and $\sigma_{DIS}$ has little difference between the two samples.

Therefore, the difference in yield strength is more likely associated with the relative contributions of solid-solution strengthening and precipitation strengthening. According to the matrix composition analysis in Table 2, only a limited amount of solute is retained in the matrix in both conditions, suggesting that the difference in solid-solution strengthening is relatively small. Overall, precipitation strengthening is considered to be the dominant strengthening mechanism in the present alloy after post-processing.

### 3.6. Fractography

**Figure 9:**
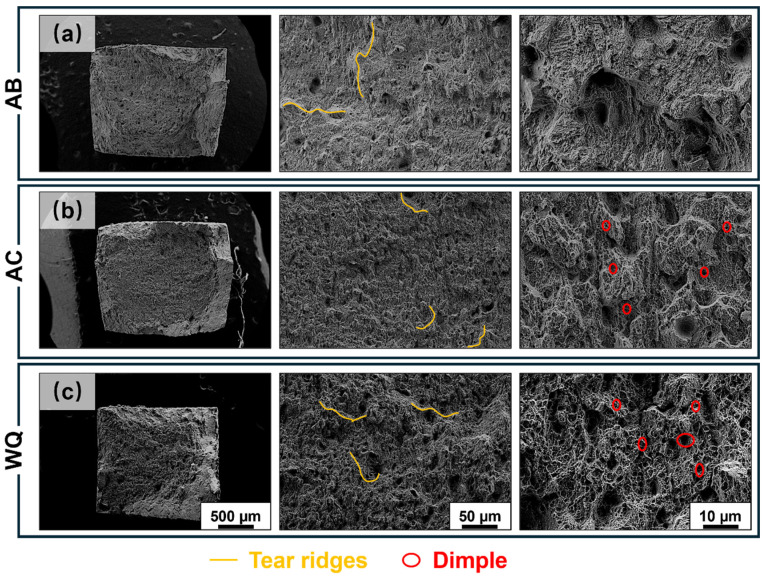
SEM fractography of the tensile specimens for the (**a**) AB, (**b**) AC, and (**c**) WQ samples at different magnifications.

Figure 9 presents the fracture morphologies of the tensile specimens under the AB, AC, and WQ conditions. Clear differences in fracture topography are observed among the three samples, which are in good agreement with their respective tensile responses.

The AB sample (Figure 9a) exhibits a relatively flat fracture surface containing shallow dimples together with noticeable tear ridges. This morphology suggests a mixed fracture mode in which ductile microvoid deformation is present, but the plastic deformation capability remains moderate. In other words, the AB specimen does not fail by purely brittle cleavage, yet its fracture surface indicates that plastic strain accommodation before final failure is still limited compared with the WQ condition. The AC sample (Figure 9b) shows a fracture surface characterized by tear ridges, shallow dimples, and locally identifiable cleavage-like facets. Such a morphology is typical of a mixed brittle–ductile fracture mode. This feature is consistent with the tensile result that the AC sample possesses the highest strength but relatively limited elongation. The presence of cleavage-like regions suggests that local stress concentration during deformation is still significant, which may be associated with the stronger overall hardening state and the relatively coarser grain-boundary borides formed during slower cooling. By contrast, the WQ sample (Figure 9c) exhibits a much more tortuous fracture surface with a high density of dimples. The dimples are more numerous and better developed than those observed in the AB and AC samples, indicating a greater extent of plastic deformation prior to fracture. Although a few isolated cleavage-like facets can still be observed locally, the overall fracture morphology is clearly dominated by ductile features. This implies that microvoid nucleation, growth, and coalescence are the primary fracture mechanisms in the WQ sample, which is consistent with its significantly enhanced tensile elongation.

The transition in fracture behavior from AB and AC to WQ can be understood in connection with the preceding microstructural observations. As shown in Figure 4, post-deposition heat treatment breaks up the continuous brittle segregation network in the AB state, thereby reducing the continuity of crack-sensitive paths. Furthermore, the WQ sample retains a finer and denser grain-boundary particle distribution than the AC sample, which is expected to alleviate local stress concentration and delay crack initiation and propagation. In contrast, the relatively coarser boundary particles in the AC condition may promote local crack nucleation more readily during tensile loading, leading to a stronger tendency toward quasi-cleavage fracture. Therefore, the fracture morphologies provide further support for the conclusion that the distinct tensile behaviors of AC and WQ arise from different balances between boundary damage tolerance and overall strengthening state.

## 4. Conclusions

The effects of post-process cooling strategies on the microstructure and mechanical performance of an LPBF-fabricated Fe-based alloy were systematically investigated. The primary conclusions are as follows:

(1) A novel Fe-based alloy, designated as AMSD, was successfully designed through a machine-learning-assisted high-throughput alloy design strategy and fabricated by LPBF without obvious solidification cracking. The alloy exhibited excellent processability over a relatively broad processing window. In the as-built state, the microstructure consisted of a typical rapidly solidified cellular structure and epitaxial columnar grains, accompanied by pronounced intercellular segregation and a continuous network-like phase enriched in strong carbide-forming elements.
(2) After post-processing, the continuous intercellular segregation network in the as-built state was eliminated, while discrete Mo-W-Ti-rich grain-boundary borides were clearly observed in the AC and WQ samples. Microstructural evolution was strongly governed by cooling kinetics: the slower air-cooling (AC) route allowed more extensive coarsening of grain-boundary borides, whereas water quenching (WQ) retained a finer boride population by suppressing further diffusional growth.
(3) The mechanical properties of the AMSD alloy can be effectively tailored by controlling the cooling rate after solution treatment. The air-cooled sample exhibits an ultra-high UTS of 1436 ± 45 MPa with moderate ductility (9.4 ± 0.4%), whereas the water-quenched sample demonstrates significantly improved elongation (18.2 ± 0.3%) with a UTS of 1072 ± 15 MPa, achieving an excellent strength–ductility balance.

## Figures and Tables

**Table 1:** Chemical composition of AMSD powder (wt.%).

Alloy	Cr	Ni	Mo	W	Al	Ti	C	B	Si	Mn	Fe
AMSD	10.06	37.6	1.96	1.72	1.99	4.90	0.072	0.17	0.38	0.26	Bal.

**Table 2:** Results of EDS point analysis for the area marked in Figure 4c,f,i (wt.%).

Point	Cr	Ni	Mo	W	Al	Ti	C	B	Si	Fe
A	8.29	35.25	0.85	3.71	2.45	5.20	6.79	0	0.05	37.4
B	7.34	38.35	2.46	2.04	2.09	4.64	7.1	0	1.27	34.7
C	10.96	27.96	4.03	3.86	1.35	5.68	9.94	0.17	0.05	35.2
D	7.04	35.22	3.06	1.65	1.41	2.92	5.98	0.85	0.14	41.48
E	7.82	3.81	15.22	29.11	0.19	11.4	3.91	14.19	0.58	13.73
F	9.37	33.55	2.98	2.43	1.48	2.10	4.11	0.74	0.77	42.11
G	9.55	33.51	2.69	2.48	2.16	3.47	5.63	1.14	0.19	38.03
H	7.89	4.53	18.94	27.83	0.23	12.70	4.15	14.70	0.24	8.53
I	9.44	36.66	1.42	1.79	2.53	3.62	4.49	0.74	0.60	38.39

## Data Availability

The original contributions presented in this study are included in the article. Further inquiries can be directed to the corresponding author.
